# Nationwide Screening for Bee Viruses and Parasites in Belgian Honey Bees

**DOI:** 10.3390/v12080890

**Published:** 2020-08-14

**Authors:** Severine Matthijs, Valérie De Waele, Valerie Vandenberge, Bénédicte Verhoeven, Jacqueline Evers, Marleen Brunain, Claude Saegerman, Paul J. J. De Winter, Stefan Roels, Dirk C. de Graaf, Nick De Regge

**Affiliations:** 1 Belgian National Reference Laboratory for Bee Diseases, Unit of Enzootic, Vector-Borne and Bee Diseases, Sciensano, Juliette Wytsmanstraat 14, 1050 Brussels, Belgium; valerie_vandenberge@yahoo.com (V.V.); Stefan.Roels@dgz.be (S.R.); Nick.DeRegge@sciensano.be (N.D.R.); 2 Veterinary Epidemiology, Sciensano, Juliette Wytsmanstraat 14, 1050 Brussels, Belgium; valerie.dewaele@spw.wallonie.be; 3 Federal Agency for the Safety of the Food Chain, Kruidtuinlaan 55, 1000 Brussels, Belgium; BENEDICTE.VERHOEVEN@favv-afsca.be (B.V.); Jacqueline.Evers@afsca.be (J.E.); PAUL.DEWINTER@favv-afsca.be (P.J.J.D.W.); 4 Laboratory of Molecular Entomology and Bee Pathology, Ghent University, Krijgslaan 281 S2, 9000 Ghent, Belgium; Marleen.Brunain@UGent.be (M.B.); Dirk.deGraaf@UGent.be (D.C.d.G.); 5 Research Unit of Epidemiology and Risk Analysis Applied to Veterinary Sciences, Fundamental and Applied Research for Animal and Health (FARAH) Center, University of Liège, Quartier Vallée 2, Avenue de Cureghem 7A B42, 4000 Liège, Belgium; claude.saegerman@ulg.ac.be

**Keywords:** honey bee virus, honey bee parasite, virus screening, Belgium, Varroa, Nosema, DWV

## Abstract

The health of honey bees is threatened by multiple factors, including viruses and parasites. We screened 557 honey bee (*Apis mellifera*) colonies from 155 beekeepers distributed all over Belgium to determine the prevalence of seven widespread viruses and two parasites (*Varroa* sp. and *Nosema* sp.). Deformed wing virus B (DWV-B), black queen cell virus (BQCV), and sacbrood virus (SBV) were highly prevalent and detected by real-time RT-PCR in more than 95% of the colonies. Acute bee paralysis virus (ABPV), chronic bee paralysis virus (CBPV) and deformed wing virus A (DWV-A) were prevalent to a lower extent (between 18 and 29%). Most viruses were only present at low or moderate viral loads. Nevertheless, about 50% of the colonies harbored at least one virus at high viral load (>10^7^ genome copies/bee). *Varroa* mites and *Nosema* sp. were found in 81.5% and 59.7% of the honey bee colonies, respectively, and all *Nosema* were identified as *Nosema ceranae* by real time PCR. Interestingly, we found a significant correlation between the number of *Varroa* mites and DWV-B viral load. To determine the combined effect of these and other factors on honey bee health in Belgium, a follow up of colonies over multiple years is necessary.

## 1. Introduction

The ecological and economic importance of honey bees is well established. Honey bees are highly valuable pollinators for both wild flowering plants and economically important crops. However, honey bees and other pollinators face multiple threats, including viruses, parasites, bacteria, pesticides and lack of sufficient or high-quality food. In recent years, the loss of managed colonies of the western honey bee *Apis mellifera* has prompted researchers to study the drivers of colony mortality [1,2,3,4,5]. Viruses are considered to be among the key players of honey bee declines [6,7,8,9,10]. Several of the most common honey bee viruses (deformed wing virus complex, black queen cell virus, sacbrood virus and the acute bee paralysis complex) have been found on all continents [11].

Most known honey bee viruses are positive-sense single stranded RNA viruses. Bee viruses can infect all developmental stages of the bee i.e., eggs, brood (larvae and pupae), and adults. Viruses can cause a range of clinical signs, including wing deformities, hairless bees, yellow dead larvae in the cells of worker-bees or in queen cells, and behavioral disorders such as trembling, paralysis and disorientation. However, viruses generally persist naturally in bee populations at low viral loads, without apparent clinical signs (covert infection), although they might shorten the life span of the bees [12].

Because honey bees are social insects, living in large colonies consisting of a queen and several thousand of her offspring (up to 80,000 worker bees and several hundred drones), viruses can easily be transmitted within a colony. Honey bee viruses can be transmitted vertically, from an infected queen to her offspring, and horizontally, for example, when infected worker bees feed larvae or tend to the queen, through trophallaxis, through physical contact between bees in the hive or in the field, or through a vector, like the *Varroa destructor* mites [13]. Infected drones can also pass on virus when mating with a queen [14].

*V. destructor* mites are exogenous parasites feeding on brood and adult bees, thereby causing physical damage and deformities and weakening the bees and brood. Furthermore, *V. destructor* also act as important vectors for several bee viruses [15,16,17,18,19,20]. *V. destructor* mites are found in honey bee colonies on every continent except Australia [21]. Since *V. destructor* feed on fat body tissue [22] and thereby inoculate viruses directly in the haemocoel, this results in higher mortality than when viruses are transmitted between bees by contact or ingestion [23].

The intracellular microsporidian parasites *Nosema apis* and *Nosema ceranae* are found in *A. mellifera* throughout the world and are transmitted when bees ingest the spores of these fungi (e.g. through contaminated water or food or through grooming). *N. apis* and *N. ceranae* both invade the epithelial cells of the midgut of honey bees [24]. *N. ceranae* has been shown to not only damage the midgut epithelial cells but also suppress the honeybee immune response, which could lead to an increased severity of the effects of the viral pathogens [25]. Both synergistic and antagonistic interactions have been suggested between *Nosema* sp. infection and virus infection [25,26,27].

**Figure 1:**
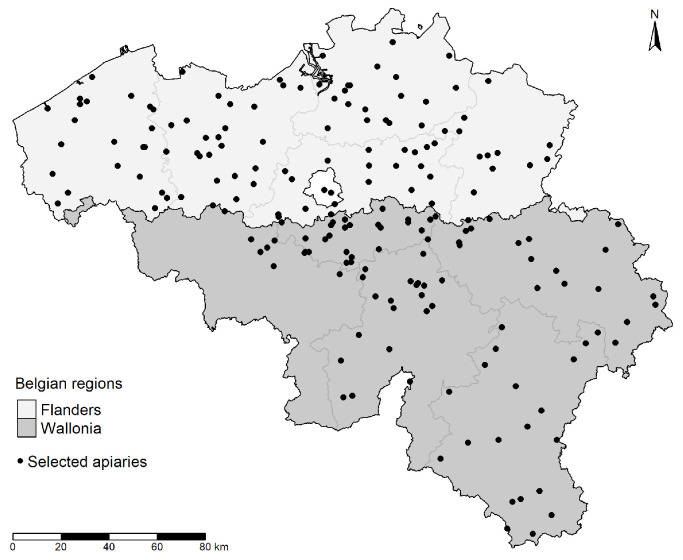
Map of Belgium and its provinces showing the locations of the selected apiaries (black dots). Provinces in Flanders in light grey, provinces of Wallonia in dark grey.

Two earlier virus screenings have been performed in Belgium in honey bees from Wallonia (southern Belgium) in 2006 [28] and Flanders (northern Belgium) in 2011 [29]. The most common virus in Wallonia was black queen cell virus (found in 75% of the 36 sampled apiaries), followed by chronic bee paralysis virus and sacbrood virus. In Flanders, viruses from the deformed wing virus complex were found in almost 70% of the colonies from 170 beekeepers. In order to gain insight into the change in virus prevalence over time, we performed a large scale screening in honey bees collected in spring 2017 from 557 colonies belonging to 155 apiaries, homogeneously distributed over Belgium (Figure 1). The bees were used to determine the prevalence and viral load of seven widespread honey bee viruses (acute bee paralysis virus (ABPV), chronic bee paralysis virus (CBPV), black queen cell virus (BQCV), deformed wing virus A (DWV-A), deformed wing virus B (DWV-B), Kashmir bee virus (KBV) and sacbrood virus (SBV)) and the prevalence of *Nosema* sp.. We furthermore analyzed whether a link could be found between virus prevalence or viral load and *V. destructor* abundance in the same colonies during autumn 2016.

## 2. Materials and Methods

### 2.1. Honey Bee Sampling

A two-stage random sampling strategy was chosen, with 20 apiaries per province, and up to 8 hives per apiary. In autumn 2016, 193 selected apiaries were visited by inspectors from the Belgian Federal Agency for the Safety of the Food Chain (FASFC) to assess the number of *V. destructor* mites (and other colony attributes). In the spring of 2017, 155 of these apiaries, holding 557 honey bee colonies, were sampled for the detection of viruses and *Nosema* sp.. For these analyses, about 100 adult honey bees (*Apis mellifera*) were collected from each of the 557 colonies and stored at −20 °C until further processing. Although samples collected in this study were kept at −20 °C, preservation of samples at −80 °C is preferable to reduce viral degradation. Storage at −20 °C might lead to an under-estimation of viral prevalence [30].

### 2.2. Virus Detection

Five adult worker bees from each of the 557 colonies were homogenized in 2.5 mL tissue lysis buffer (ATL, Qiagen, Hilden, Germany) using a ribolyser at the Belgian National Reference Laboratory for Bee diseases, Sciensano. After centrifugation, DNA and RNA were extracted from 115 µL supernatant with the MagMAX Express (Indical Bioscience, Leipzig, Germany) using the MagMAX™ Pathogen RNA/DNA Kit (Applied Biosystems, Foster City, CA).

For the virus detection, 5 µL of the extract was used in a one-step RT-rtPCR with AgPath-ID™ One-Step RT-PCR Reagent (Applied Biosystems) on a LightCycler 480 II instrument (Roche Life Science, Penzberg, Germany). The primers and probes used are listed in Table 1. The following amplification cycle was used: 10 min at 45 °C, 10 min at 95 °C, followed by 45 cycles of 15 s at 95 °C and 1 min at 60 °C.

Negative extraction controls and negative amplification controls were added to each run. Three dilutions of a DNA plasmid containing the target sequence of each virus were added to every run to provide an estimate of the amount of viral RNA present in the samples. The added dilutions were V5 (10^5^ viral genome copies per 5 µL, equivalent to 10^7^ copies/bee), V4 (10^4^ viral genome copies per 5 µL, equivalent to 10^6^ copies/bee) and V2 (10^2^ viral genome copies per 5 µL, equivalent to 10^4^ copies/bee). Samples were identified as “strong positive” for a virus if the threshold cycle (Ct value) was lower than the threshold cycle of the V5 dilution of the plasmid, i.e., if more than 10^7^ genome copies/bee were detected. Samples with less than 10^4^ genome copies/bee (Ct value higher than the Ct value of V2) were identified as “weakly positive”. Samples with intermediate results were labelled “moderately positive”.

### 2.3. Varroa Mite Counts

*V. destructor* mites were counted by trained inspectors of the FASFC in autumn 2016 using the powdered sugar method. About 100 bees were shaken from a comb and placed in a pot, weighed (to estimate the number of bees) and powdered sugar was added. After about three minutes with regular gentle shaking, the *V. destructor* that fell off the bees were counted and averaged as number of mites per 100 bees.

### 2.4. Nosema Spore Counts and Species Identification

The abdomens of 60 adult worker bees from each of the 557 sampled colonies were homogenized in 8 mL PBS using a bullet blender. An amount of 100 µL of sample was further diluted in PBS to obtain a dilution of 1 mL PBS per bee. Spores were counted in 18 squares in both chambers of a Bürker counting chamber at the Laboratory of Molecular Entomology and Bee Pathology, Ghent University. Counts were converted to the number of spores per bee. Spore counts below 20,000 spores per bee were labelled as “not detected” (threshold of detection).

To determine whether the honey bees were infected with *Nosema apis* or *Nosema ceranae*, 5 µL of the DNA/RNA extract (same extract as used for virus detection, see Section 2.2) was used in a real time PCR using the SsoAdvanced Universal Probes Supermix (BioRad, Hercules, CA) with species specific primers and probes (Table 2) with the following amplification cycle: 2 min at 95 °C, followed by 45 cycles of 15 s at 94 °C, 30 s at 55 °C and 30 s at 68 °C. Negative extraction controls and positive and negative amplification controls were added to each run.

### 2.5. Statistical Analysis

Fisher exact tests were performed in GraphPad Instat 3 (San Diego, CA) to determine if there were significant differences between the prevalence of the different viruses between Flanders and Wallonia. A linear regression analysis was performed using the data analysis tools provided by Excel (Microsoft, Redmond, WA) to analyze whether the viral load of the different viruses present in a colony was correlated with the number of *V. destructor* mites (per 100 bees) in those colonies. *p* values lower than 0.05 were considered to be significant.

## 3. Results

### 3.1. Virus Prevalence and Viral Load

**Figure 2:**
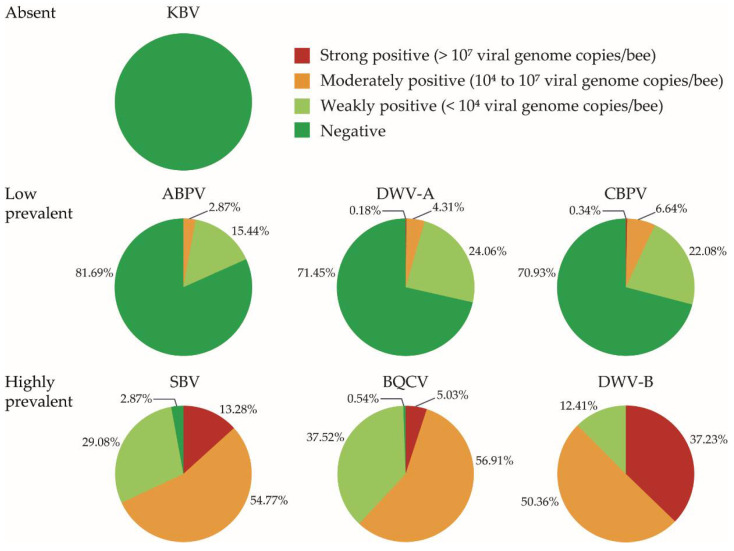
Prevalence and viral load of 7 viruses in 557 colonies of 155 apiaries homogeneously distributed over Belgium.

Viral RNA was detected in all screened colonies. The seven viruses we screened for can be categorized as absent, low prevalent or highly prevalent in Belgian managed honey bee colonies (Figure 2). KBV was not detected in any of our samples, while ABPV, CBPV and DWV-A were found with a low prevalence (between 18% and 29% of the colonies). Three viruses were found with a high prevalence: BQCV and SBV were present in more than 95% of the analyzed honey bees, while DWV-B was detected in every analyzed sample. However, for most of the viruses, colonies were generally weakly or moderately positive, except for DWV-B for which high viral loads were found in 37% of the colonies (Figure 2).

When analyzing the data from apiaries from Flanders and Wallonia separately (Table 3), we found a significant difference between the prevalence of ABPV and DWV-A between both regions. In Flanders, 8.8% of the colonies were infected with ABPV, while 26.5% of the colonies from Wallonia were positive for ABPV (Fisher exact test; *p* < 0.001). DWV-A was detected in 18% of the Flemish colonies and in 37.75% of the colonies in Wallonia (Fisher exact test; *p* < 0.001). The differences between the regions for the other viruses were not significant (Fisher exact test; *p* > 0.05).

**Figure 3:**
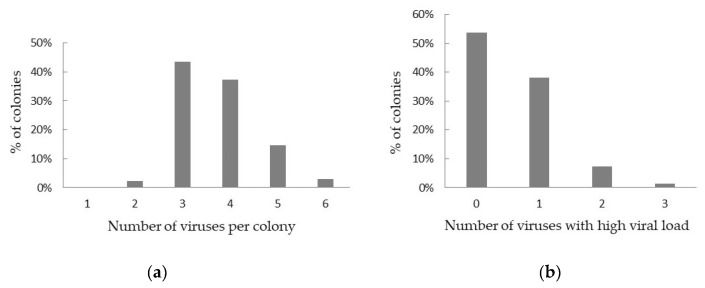
Viral co-infection of Belgian honey bee colonies. (**a**) Percentage of honey bee colonies infected with multiple viruses; (**b**) percentage of colonies infected with zero, one or several viruses at high viral load.

All screened colonies were infected with at least two viruses (Figure 3a). Twelve colonies were infected with only two viruses, 11 of these with DWV-B and BQCV. In almost half of the colonies (43.27%), three different viruses were detected, most often DWV-B, BQCV and SBV (236 out of 241 colonies). Sixteen colonies (2.87%) were infected with six different viruses. However, in more than half of all colonies (53.5%), only infections with low viral RNA loads were detected (Figure 3b). The maximum number of viruses with a high viral load found in one colony was 3 (in 7 colonies, each time BQCV, DWV-B and SBV).

### 3.2. Varroa Prevalence and Abundance

*V. destructor* mites were found in 454 out of 540 honey bee colonies (81.5% of the colonies, 94.84% of the apiaries) (17 colonies with missing data). However, the majority of the colonies (79.35%) had a low or very low infestation level (less than 10 mites per 100 bees). Only one colony had a high infestation with *V. destructor* (more than 50 mites per 100 bees).

### 3.3. Correlation between Virus Load and Varroa Infestation

We found a significant positive linear relationship between the number of *V. destructor* mites and the viral load of DWV-B present in a colony (linear regression analysis; *p*-value of the slope = 0.0051). There was no significant correlation between the number of *V. destructor* in the colonies and the viral load of the other viruses (linear regression analysis; *p*-value > 0.05).

### 3.4. Nosema Prevalence, Abundance and Species Identification

*Nosema* sp. spores were detected in 59.7% of the honey bee samples (from 20,103 spores/bee to 51,106 spores/bee). Real time PCR identified all *Nosema* sp. as *N. ceranae*.

## 4. Discussion

The main objective of this study was to obtain insights into the current prevalence of seven honey bee viruses in Belgian honey bees and to evaluate how this prevalence changed during the past decade. We found nearly all 557 sampled honey bee colonies to be infected with BQCV (99.46%), DWV (DWV-A in 28.55% and DWV-B in 100% of the colonies) and SBV (91.13% of the colonies). Our results seem to indicate a strong increase in the prevalence of honey bee viruses in Belgium over the last decade. In 2006, BQCV was detected in 75% of the sampled apiaries in the southern part of Belgium (Wallonia), while SBV and DWV (without making a distinction between DWV-A or DWV-B) were detected in 69% and 64% of the apiaries, respectively [28]. In bees sampled in 2011 in the northern part of Belgium (Flanders), BQVC and SBV were only detected in 13.5% and 19% of the colonies, respectively, and DWV was the most prevalent virus, found in 69.4% of the sampled colonies (without making a distinction between DWV-A or DWV-B) [29]. We found ABPV to be prevalent at a lower rate, but still an increased prevalence compared to previous screenings was noted: we detected ABPV in 18% of the colonies, while it was found in 8% of the apiaries in Wallonia in 2006 and 3.3% of the colonies in Flanders in 2011. When we looked at our data from apiaries from Flanders and Wallonia separately (Table 3), we found a significantly higher prevalence of ABPV in our samples from Wallonia (26.5%) compared to Flanders (8.8%) (Fisher exact test; *p* < 0.001). The situation was different for CBPV, where we detected the virus in 29% of the colonies, which is an important increase compared to the 1.7% found in Flanders in 2011, but a considerable decrease compared to the 69% found in Wallonia in 2006 [28]. We found no significant difference in the number of CBPV infected colonies in both regions (29.23% in Flanders and 30.27% in Wallonia) (Fisher exact test; *p* > 0.05). KBV was not detected in any of the colonies during our screening and had not been looked for in the previous screenings. In a multi-year survey in the United States (2009–2014), a strong increase in virus prevalence was also observed over the years, with DWV prevalence rising from 65% to over 80% and the number of BQCV positive samples climbing from 60% to 90% in only three years’ time [39]. Moreover, as in previous studies, seasonal variation in virus prevalence was observed [12,39]. A small part of the differences we observe between our screening and previous studies might be related to seasonal variation. Additionally, it should be considered that the higher number of virus-infected colonies in our study compared to earlier studies might for a small part be due to a possibly higher sensitivity of the real time PCR method used. The two previous studies used either traditional reverse transcription PCR [28] or MLPA (multiplex ligation probe dependent amplification) [29] to detect selected honey bee viruses, resulting only in presence/absence data without information on the viral load. 

Overall, our results not only reflect an increase in the prevalence of honey bee viruses but also an expansion of the distribution area of viruses over the years within the country. On an international scale, dissemination of honey bee viruses is considered to be caused in part by colony transportation (migratory beekeeping) and international trade of (infected) queens and honey bee packages [11]. However, few Belgian beekeepers move their colonies and import of queens is very limited, although viruses have also been found in eggs from a Belgian queen breeding program [40]. Viruses can also be imported in a hive through contaminated material or bee products [41,42,43]. Spread of viruses can furthermore happen through contact with contaminated honey bees from other hives while foraging, but also through contact with other infected insects, as honey bee viruses have been detected in other insect pollinators, including bumble bees and hoverflies [44,45,46]. *V. destructor* mites are another factor that could be implicated in the extended virus spread, by acting as a vector for several viruses. *V. destructor* mites were found in 81.5% of the colonies during the autumn preceding the sampling for screening of viruses, although the infestation rate was rather low (mostly less than 10 mites per 100 bees), likely owing to the Varroa management practices of the beekeepers. However, we did find a significant correlation between the number of mites and the viral load of DWV-B (linear regression analysis; *p*-value < 0.05). This is in line with findings from other studies that not only show that *V. destructor* is a vector of DWV-B (and other viruses), but that it can also suppress honey bee immunity, resulting in higher viral loads [16,17,20,47].

The high prevalence of these viruses in Belgium is also in line with prevalence rates reported in neighboring countries. A screening of seemingly healthy honey bees from France in 2002 already revealed DWV to be present in 97% of the 36 sampled apiaries, while BQCV and SBV were detected in 86% of the apiaries [48]. In Germany, DWV was found in 33.4% of 120 apiaries in 2007 [2]. In the Netherlands, to the north of Belgium, DWV has been detected in 93% of 331 investigated honey bee colonies in 2015 [49]. Interestingly, KBV was found in 17% of French apiaries already in 2002 [48], while we did not detect this virus at all. It would be worthwhile to screen honey bee colonies at the Belgian–French border for KBV. Analogous to our results, other virus screenings also report honey bee colonies to be infected with multiple viruses, with, e.g., 92% of the French apiaries positive for at least three different viruses [6,28,48,50]. We found more than 95% of our colonies to be infected with three or more viruses. This underlines the importance of including co-infection with multiple viruses in future studies on bee health and colony losses.

Although the prevalence of viruses was high among these Belgian honey bees, it is important to note that most of the colonies were only weakly or moderately positive for a virus (see Figure 2), so it cannot be deduced that these viruses have a strong impact on bee health. High viral loads for one or several viruses were however found in about 46% of the colonies. Although colonies displaying clinical signs usually have high viral loads, a high viral load does not necessarily result in clinical signs. Thresholds between 10^5^ and 10^10^ viral genome copies per bee have been suggested for different honey bee viruses to distinguish between covert and overt infections, but not all colonies with a viral load above the defined thresholds in these studies displayed clinical signs [34,36,50]. This is especially the case for DWV-B, where 45% of the colonies with a viral load above the defined threshold did not display clinical signs [34]. The appearance of clinical signs depends on many factors. Sometimes the occurrence of some clinical signs can be “hidden” by the hygienic behavior of the bees, when larvae or pupae with disease symptoms are removed from the colony [34]. The virulence of honey bee viruses is also influenced by the transmission route or type of tissue or life-stage that becomes infected [12]. On the other hand, even covert infections can still influence honey bee survival, and several viruses have been linked with overwintering colony mortality even in the absence of (visible) clinical signs [51,52,53].

Co-infections with other stress factors such as *V. destructor* and *Nosema* sp. can aggravate disease symptoms. Interesting in this perspective was our finding that more than half of the samples were found to be infected with *Nosema* sp.. It is noteworthy that only *N. ceranae* was detected in our samples, while a previous study on samples from 2011 in Belgium found about 10% of *Nosema*-infected samples to be *N. apis* [54]. This indicates that *N. ceranae* might have replaced *N. apis* in Belgium, a trend that has already been observed in other countries [3,55] and could be caused by the inhibition of *N. apis* by *N. ceranae* in case of co-infection [56].

In conclusion, we found a high prevalence of viruses in Belgian managed honey bee colonies, but with a highly variable viral load. Additionally, we confirmed the presence of other harmful factors such as *V. destructor* and *N. ceranae* in more than half of the sampled colonies. This accentuates the importance of identifying co-infections with multiple pathogens when studying honey bee health. A follow up of colonies over multiple years would be necessary to determine the contribution of these combined factors to colony losses in Belgium.

## Figures and Tables

**Table 1:** Primers and probes used for real time PCR detection of viruses.

Virus	Sequences Primers and Probe (5′–3′)	Reference	Final Primer—Probe Concentration
ABPV	Forward CATATTGGCGAGCCACTATG	[31]	800 nM—100 nM
	Reverse CTACCAGGTTCAAAGAAAATTTC		
	Probe ATAGTTAAAACAGCTTTTCACACTGG		
BQCV	Forward GGTGCGGGAGATGATATGGA	[32]	320 nM—200 nM
	Reverse GCCGTCTGAGATGCATGAATAC		
	Probe TTTCCATCTTTATCGGTACGCCGCC		
CBPV	Forward CGCAAGTACGCCTTGATAAAGAAC	[33]	320 nM—200 nM
	Reverse ACTACTAGAAACTCGTCGCTTCG		
	Probe TCAAGAACGAGACCACCGCCAAGTTC		
DWV-A	Forward GCGGCTAAGATTGTAAATTG	[34]	350 nM—100 nM
	Reverse GTGACTAGCATAACCATGATTA		
	Probe CCTTGACCAGTAGACACAGCATC		
DWV-B	Forward GGTCTGAAGCGAAAATAG	[34]	600 nM—200 nM
	Reverse CTAGCATATCCATGATTATAAAC		
	Probe CCTTGTCCAGTAGATACAGCATCACA		
KBV	Forward ACCAGGAAGTATTCCCATGGTAAG	[35]	500 nM—200 nM
	Reverse TGGAGCTATGGTTCCGTTCAG		
	Probe CCGCAGATAACTTAGGACCAGATCAATCACA		
SBV	Forward AACGTCCACTACACCGAAATGTC	[36]	320 nM—200 nM
	Reverse ACACTGCGCGTCTAACATTCC		
	Probe TGATGAGAGTGGACGAAGA		
actin	Forward AGGAATGGAAGCTTGCGGTA	[37]	500 nM—200 nM
	Reverse AATTTTCATGGTGGATGGTGC		
	Probe ATGCCAACACTGTCCTTTCTGGAGGTA		

**Table 2:** Primers and probes used for real time PCR detection of *Nosema apis* or *Nosema ceranae*.

*Nosema* Species	Sequences Primers and Probe (5′–3′)	Reference	Final Primer—Probe Concentration
*Nosema apis*	Forward CCATTGCCGGATAAGAGAGT	[38]	300 nM—150 nM
	Reverse CCACCAAAAACTCCCAAGAG		
	Probe ATAGTGAGGCTCTATCACTCCGCTG		
*Nosema ceranae*	Forward CGGATAAAAGAGTCCGTTACC	[38]	300 nM—150 nM
	Reverse TGAGCAGGGTTCTAGGGAT		
	Probe CGTTACCCTTCGGGGAATCTTC		

**Table 3:** Percentages of positive colonies per virus in honey bee colonies from Flanders and Wallonia and indications of increase (+) or decrease (-) compared to previous studies.

Virus	Percentage of Positive Colonies in Flanders	Trend ^1^	Percentage of Positive Colonies in Wallonia	Trend ^2^
ABPV	8.8%	+	26.5%	+
BQCV	99.23%	+	99.65%	+
CBPV	29.23%	+	30.27%	-
DWV-A	18.07%	+ ^3^	37.75%	+ ^3^
DWV-B	100%		100%	
KBV	0	no data	0	no data
SBV	97.69%	+	96.26%	+

^1^ Compared to samples taken in Flanders in 2011 [29]. ^2^ Compared to samples taken in Wallonia in 2006 [28]. ^3^ No distinction was made between DWV-A and DWV-B in these previous studies.
